# Illicitly Manufactured Fentanyl–Involved Overdose Deaths with Detected Xylazine — United States, January 2019–June 2022

**DOI:** 10.15585/mmwr.mm7226a4

**Published:** 2023-06-30

**Authors:** Mbabazi Kariisa, Julie O’Donnell, Sagar Kumar, Christine L. Mattson, Bruce A. Goldberger

**Affiliations:** ^1^Division of Overdose Prevention, National Center for Injury Prevention and Control, CDC; ^2^Forensic Medicine Division, Department of Pathology, Immunology and Laboratory Medicine, College of Medicine, University of Florida, Gainesville, Florida.

In 2022, provisional data indicated that more than two thirds (68%) of the reported 107,081 drug overdose deaths in the United States involved synthetic opioids other than methadone, principally illicitly manufactured fentanyls (IMFs) (*1*). Xylazine, a nonopioid sedative not approved for human use and with no known antidote, has been increasingly detected in IMF products in the U.S. drug supply* and in IMF-involved overdose deaths (*2*). Limited studies suggest xylazine can cause central nervous system depression, respiratory depression, bradycardia, and hypotension in humans (*3*,*4*); chronic use might lead to severe withdrawal symptoms^†^ as well as skin ulcerations (*4*). This report uses data from CDC’s State Unintentional Drug Overdose Reporting System (SUDORS) to describe IMF-involved^§^ overdose deaths with and without xylazine detected that occurred during January 2019–June 2022. Among 21 jurisdictions, which included 20 states and the District of Columbia, the monthly percentage of IMF-involved deaths with xylazine detected increased 276%, from 2.9% to 10.9%. Among IMF-involved deaths during January 2021–June 2022 in 32 jurisdictions, xylazine was detected in a higher percentage of jurisdictions in the Northeast U.S. Census Bureau region; listing detected xylazine as a cause of death varied across jurisdictions. Expanded postmortem and illicit drug product testing for xylazine is needed to clarify prevalence in drug supplies; further investigation of xylazine’s effects on humans is necessary to characterize morbidity and overdose risk. It is important for overdose prevention and response messages to highlight the potential presence of xylazine in IMF products and emphasize the need for respiratory and cardiovascular support to address the sedative effects of xylazine.

Jurisdictions entered information on drug overdose deaths that were unintentional or of undetermined intent into SUDORS using death certificates, medical examiner and coroner reports (including information about circumstances of the overdose from scene evidence and witness reports), and toxicology reports. Monthly counts of IMF-involved deaths^¶^ with xylazine detected and co-involved as a cause of death, and proportions of IMF-involved deaths with xylazine detected were examined in 21 jurisdictions** for January 2019–June 2022. The most recent 18 months of data (January 2021–June 2022) were further examined among 32 jurisdictions.^††^ The number and percentage of IMF-involved deaths with xylazine detected, and the proportion of those with xylazine detected for which xylazine was listed as a cause of death, were calculated for each jurisdiction. The number and percentage of IMF-involved deaths with and without xylazine detected were calculated, stratified by decedent demographics, U.S. Census Bureau region,^§§^ co-involved drugs, and overdose circumstances (e.g., route of drug use, decedent drug use history, and overdose response efforts). Jurisdictions were included in analyses if toxicology reports were available for ≥75% of deaths for the relevant study periods, resulting in variation in the number of states included in each analysis; analyses were restricted to deaths with toxicology reports or with xylazine listed as a cause of death on the death certificate. Analyses were performed using SAS (version 9.4; SAS Institute). This activity was reviewed by CDC and conducted consistent with applicable federal law and CDC policy.^¶¶^

Among 21 jurisdictions, the monthly proportion of IMF-involved deaths with xylazine detected increased 276% from January 2019 (2.9%) to June 2022 (10.9%) (Figure 1). The monthly number of IMF-involved deaths with xylazine co-involved increased from 12 in January 2019 to 188 in June 2022. During January 2021–June 2022, among 32 jurisdictions, xylazine was detected in 9.0% (4,859) of 53,969 IMF-involved deaths (Table) and co-involved in 6.9% (3,735). Xylazine detection varied by jurisdiction (Figure 2). The highest percentages and numbers of IMF-involved deaths with xylazine detected were in Maryland (27.7%; 923 deaths), Connecticut (26.4%; 507), and Pennsylvania (23.3%; 1,285). The proportion of IMF-involved deaths with xylazine detected in which xylazine was determined to be a cause of death ranged from none to ≥90% across jurisdictions. Although jurisdictions that did not submit toxicology reports to SUDORS for ≥75% of deaths were excluded from analyses, death certificate data provided to SUDORS indicated that xylazine was co-involved in IMF-involved deaths in several excluded states, including New York, which recorded 735 such deaths.***

**FIGURE 1:**
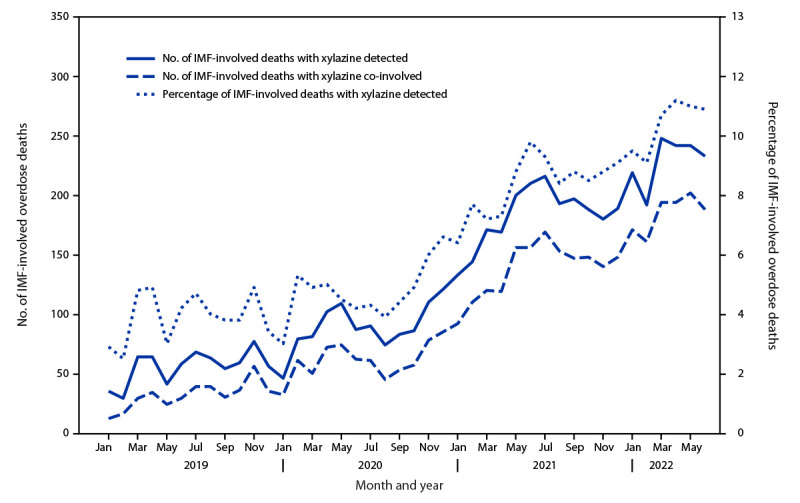
Number and percentage of drug overdose deaths involving* illicitly manufactured fentanyls,^†^ by month and xylazine detection or co-involvement — State Unintentional Drug Overdose Reporting System, 21 jurisdictions,^§^ January 2019–June 2022 **Abbreviations:** IMF = illicitly manufactured fentanyl; SUDORS = State Unintentional Drug Overdose Reporting System. * A drug was considered involved or co-involved if it was listed as a cause of death on the death certificate or medical examiner or coroner report. ^†^ Fentanyl was classified as likely illicitly manufactured using toxicology, scene, and witness evidence. For the 8% of deaths involving fentanyl that had insufficient evidence for classification as illicit or prescription, fentanyl was classified as illicit because the vast majority of fentanyl overdose deaths involve illicit fentanyl. All fentanyl analogs except alfentanil, remifentanil, and sufentanil, which have legitimate human medical use, were included as IMFs. ^§^ Connecticut, Delaware, District of Columbia, Georgia, Illinois, Maine, Massachusetts, Minnesota, Nevada, New Hampshire, New Jersey, New Mexico, Ohio, Oklahoma, Pennsylvania, Rhode Island, Utah, Vermont, Virginia, Washington, and West Virginia. Illinois, Pennsylvania, and Washington reported deaths from counties that accounted for ≥75% of drug overdose deaths in the state in 2017 per SUDORS funding requirements; all other jurisdictions reported deaths from the full jurisdiction. Jurisdictions were included if data were available for each 6-month period (January–June 2019, July–December 2019, January–June 2020, July–December 2020, January–June 2021, July–December 2021, and January–June 2022), and toxicology reports were available for ≥75% of deaths in the included period or periods. Analysis was restricted to decedents with an available toxicology report or with xylazine listed as a cause of death on the death certificate.

**TABLE:** Characteristics of illicitly manufactured fentanyl–involved* overdose decedents and circumstances surrounding death, by xylazine detection — State Unintentional Drug Overdose Reporting System, 31 states and District of Columbia,^†^ January 2021–June 2022

Characteristic	No. (%) of IMF-involved overdose deaths		
	Total	With xylazine detected	Without xylazine detected
**Overall**	**53,969 (100)**	**4,859 (9.0)**	**49,110 (91.0)**
**Sex**			
Female	**14,638 (27.1)**	1,318 (27.1)	13,320 (27.1)
Male	**39,330 (72.9)**	3,541 (72.9)	35,789 (72.9)
**Race and ethnicity^§^**			
American Indian or Alaska Native, non-Hispanic	**721 (1.3)**	13 (0.3)	708 (1.5)
Asian, non-Hispanic	**302 (0.6)**	26 (0.5)	276 (0.6)
Black or African American, non-Hispanic	**12,117 (22.6)**	1,155 (23.9)	10,962 (22.5)
Hispanic or Latino	**6,056 (11.3)**	443 (9.2)	5,613 (11.5)
Native Hawaiian or other Pacific Islander, non-Hispanic	**32 (0.1)**	0 (—)	32 (0.1)
White, non-Hispanic	**33,812 (63.1)**	3,167 (65.6)	30,645 (62.9)
Multiracial, non-Hispanic	**519 (1.0)**	26 (0.5)	493 (1.0)
**Age group, yrs**			
<15	**125 (0.2)**	10 (0.2)	115 (0.2)
15–24	**3,955 (7.3)**	229 (4.7)	3,726 (7.6)
25–34	**13,627 (25.3)**	1,200 (24.7)	12,427 (25.3)
35–44	**14,473 (26.8)**	1,338 (27.5)	13,135 (26.7)
45–54	**10,664 (19.8)**	1,007 (20.7)	9,567 (19.7)
55–64	**8,900 (16.5)**	866 (17.8)	8,034 (16.4)
≥65	**2,221 (4.1)**	209 (4.3)	2,012 (4.1)
**U.S. Census Bureau region^¶^**			
Northeast	**16,411 (30.4)**	2,423 (49.9)	13,988 (28.5)
Midwest	**15,175 (28.1)**	826 (17.0)	14,349 (29.2)
South	**14,219 (26.3)**	1,556 (32.0)	12,663 (25.8)
West	**8,164 (15.1)**	54 (1.1)	8,110 (16.5)
Co-involved drugs**			
Heroin^††^	**6,675 (12.4)**	709 (14.6)	5,966 (12.1)
Prescription opioids^§§^	**6,371 (11.8)**	695 (14.3)	5,676 (11.6)
Alcohol	**9,636 (17.9)**	724 (14.9)	8,912 (18.1)
Benzodiazepines	**6,213 (11.5)**	656 (13.5)	5,557 (11.3)
Cocaine	**16,653 (30.9)**	1,708 (35.2)	14,945 (30.4)
Methamphetamine	**11,815 (21.9)**	874 (18.0)	10,941 (22.3)
**No. of deaths with coroner or medical examiner data**	**52,684 (97.6)**	4,704 (96.8)	47,980 (97.7)
**Evidence of overdose circumstances^†^**			
Potential bystander present^¶¶^	**23,717 (45.0)**	2,128 (45.2)	21,589 (45.0)
Naloxone administration^§^	**12,442 (23.7)**	1,142 (24.3)	11,300 (23.6)
No pulse at first responder arrival^§^	**31,433 (61.4)**	2,445 (53.3)	28,988 (62.2)
Seen in the emergency department	**9,160 (18.0)**	708 (15.4)	8,452 (18.3)
History of previous overdose	**6,693 (12.7)**	546 (11.6)	6,147 (12.8)
Current or past treatment for substance use disorders	**8,066 (15.3)**	921 (19.6)	7,145 (14.9)
History of mental health diagnosis	**12,666 (24.0)**	1067 (22.7)	11,599 (24.2)
Recent release from institutional setting	**4,614 (9.1)**	528 (11.5)	4,086 (8.8)
Homelessness or housing instability	**4,712 (9.2)**	459 (10.0)	4,253 (9.2)
Route of drug use***			
Injection	**10,696 (20.3)**	1,347 (28.6)	9,349 (19.5)
Smoking	**11,402 (21.6)**	807 (17.2)	10,595 (22.1)
Ingestion	**7,411 (14.1)**	473 (10.1)	6,938 (14.5)
Snorting/Sniffing	**10,098 (19.2)**	764 (16.2)	9,334 (19.5)
Other route of drug use^†††^	**163 (0.3)**	12 (0.3)	151 (0.3)
No reported route of drug use	**24,439 (46.4)**	2,136 (45.4)	22,303 (46.5)

**Abbreviations:** IMF = illicitly manufactured fentanyl; SUDORS = State Unintentional Drug Overdose Reporting System.

* Fentanyl was classified as likely illicitly manufactured using toxicology, scene, and witness evidence. For the 8% of deaths involving fentanyl that had insufficient evidence for classification as illicit or prescription, fentanyl was classified as illicit because the vast majority of fentanyl overdose deaths involve illicit fentanyl. All fentanyl analogs except alfentanil, remifentanil, and sufentanil, which have legitimate human medical use, were included as IMFs.

^†^ Arizona, Arkansas, Colorado, Connecticut, Delaware, District of Columbia, Georgia, Illinois, Iowa, Kansas, Louisiana, Maine, Maryland, Massachusetts, Michigan, Minnesota, Nebraska, Nevada, New Hampshire, New Jersey, New Mexico, Ohio, Oklahoma, Oregon, Pennsylvania, Rhode Island, South Dakota, Utah, Vermont, Virginia, Washington, and West Virginia. Illinois, Louisiana, Pennsylvania, and Washington reported deaths from counties that accounted for ≥75% of drug overdose deaths in the state in 2017, per SUDORS funding requirements; all other jurisdictions reported deaths from the full jurisdiction. Jurisdictions were included if data were available for the full period of January 2021–June 2022, including toxicology reports for ≥75% of deaths. Analysis was restricted to decedents with an available toxicology report; or, if no toxicology report was available, deaths were also included if xylazine was listed as part of the cause of death on the death certificate. Analysis of overdose circumstances was further restricted to jurisdictions with coroner or medical examiner report data for ≥75% of deaths during January 2021–June 2022 (resulting in the same 32 jurisdictions), and to deaths with a coroner or medical examiner report.

^§^ Missing values were excluded from calculations of percentages. Percentages might not sum to 100% because of rounding.

^¶^ *Northeast:* Connecticut, Maine, Massachusetts, New Hampshire, New Jersey, Pennsylvania, Rhode Island, and Vermont; *Midwest:* Illinois, Iowa, Kansas, Michigan, Minnesota, Nebraska, Ohio, and South Dakota; *South:* Arkansas, Delaware, District of Columbia, Georgia, Louisiana, Maryland, Oklahoma, Virginia, and West Virginia; *West:* Arizona, Colorado, Nevada, New Mexico, Oregon, Utah, and Washington.

** A drug was considered involved or co-involved if it was listed as a cause of death on the death certificate or in the medical examiner or coroner report. Percentages sum to >100% because drug categories are not mutually exclusive.

^††^ Drug entries coded as heroin were heroin and 6-acetylmorphine. In addition, morphine was coded as heroin if detected along with 6-acetylmorphine or if scene, toxicology, or witness evidence indicated presence of known heroin adulterants or impurities (including quinine, procaine, xylazine, noscapine, papaverine, thebaine, or acetylcodeine), injection, illicit drug use, or a history of heroin use.

^§§^ Drug entries coded as prescription opioids were alfentanil, buprenorphine, butorphanol, codeine, dextrorphan, dihydrocodeine, hydrocodone, hydromorphone, levorphanol, meperidine, methadone, morphine, nalbuphine, noscapine, oxycodone, oxymorphone, pentazocine, prescription fentanyl, propoxyphene, sufentanil, tapentadol, and tramadol. Also included as prescription opioids were brand names and metabolites (e.g., nortramadol) of these drugs and combinations of these drugs and nonopioids (e.g., acetaminophen-oxycodone). Morphine was included as prescription only if scene or witness evidence did not indicate likely heroin use and if 6-acetylmorphine was not also detected. Fentanyl was coded as a prescription opioid based on scene, toxicology, or witness evidence.

^¶¶^ For SUDORS, a potential bystander is defined as a person aged ≥11 years who was physically nearby either during or shortly preceding a drug overdose and potentially had an opportunity to intervene or respond to the overdose. This includes any persons in the same structure (e.g., same room or same building, but different room) as the decedent during that time; a family member who was in another room during the fatal incident would be considered a potential bystander if they might have had an opportunity to provide lifesaving measures (e.g., naloxone administration), if adequate resources were available, and if they were aware that an overdose event could occur. Persons in different self-contained parts of larger buildings (e.g., a different apartment in the same apartment building) would not be considered potential bystanders.

*** Evidence of injection, smoking, snorting, ingestion, and other route of drug use are not mutually exclusive; a death could have evidence of more than one of these routes. Routes of use cannot be linked to a specific drug or type of drug.

^†††^ Other routes of drug use were buccal, sublingual, transdermal, and suppository.

**FIGURE 2:**
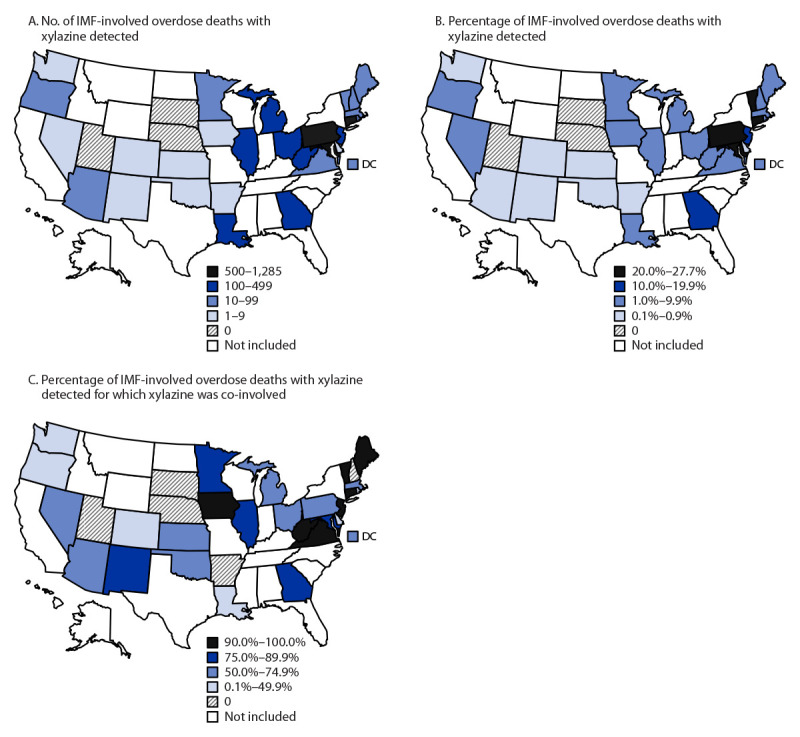
Number and percentage of drug overdose deaths involving* illicitly manufactured fentanyls,^†^ by xylazine detection or co-involvement — State Unintentional Drug Overdose Reporting System, 31 states and District of Columbia,^§^ January 2021–June 2022 **Abbreviations: **DC = District of Columbia; IMF = illicitly manufactured fentanyl; SUDORS = State Unintentional Drug Overdose Reporting System. * A drug was considered involved or co-involved if it was listed as a cause of death on the death certificate or in the medical examiner or coroner report. ^†^ Fentanyl was classified as likely illicitly manufactured using toxicology, scene, and witness evidence. For the 8% of deaths involving fentanyl that had insufficient evidence for classification as illicit or prescription, fentanyl was classified as illicit because the vast majority of fentanyl overdose deaths involve illicit fentanyl. All fentanyl analogs except alfentanil, remifentanil, and sufentanil, which have legitimate human medical use, were included as IMFs. ^§^ Arizona, Arkansas, Colorado, Connecticut, Delaware, District of Columbia, Georgia, Illinois, Iowa, Kansas, Louisiana, Maine, Maryland, Massachusetts, Michigan, Minnesota, Nebraska, Nevada, New Hampshire, New Jersey, New Mexico, Ohio, Oklahoma, Oregon, Pennsylvania, Rhode Island, South Dakota, Utah, Vermont, Virginia, Washington, and West Virginia. Illinois, Louisiana, Pennsylvania, and Washington reported deaths from counties that accounted for ≥75% of drug overdose deaths in the state in 2017, per SUDORS funding requirements; all other jurisdictions reported deaths from the full jurisdiction. Jurisdictions were included if data were available for the full period of January 2021–June 2022, including toxicology reports for ≥75% of deaths. Analysis was restricted to decedents with an available toxicology report; or, if no toxicology report was available, deaths were also included if xylazine was listed as part of the cause of death on the death certificate. Four funded states were excluded from analyses because they were known to have not tested for xylazine during the analysis period. Toxicology report data were not available for ≥75% of all deaths in eight states with complete death certificate data for January 2021–June 2022, and they were therefore excluded from analyses, but death certificate data identified IMF-involved deaths with xylazine co-involved: Alabama (46 deaths), Florida (261), Indiana (82), Mississippi (10), Missouri (93), New York (735), South Carolina (178), and Tennessee (167).

During January 2021–June 2022, decedent demographics, overdose circumstances, and other drug co-involvement were largely similar in comparisons of IMF-involved deaths with and without xylazine detected (Table). However, compared with IMF-involved deaths without xylazine, a lower percentage of those with xylazine detected had evidence of no pulse when first responders arrived (53.3% versus 62.2%) and a higher percentage had evidence of injection drug use^†††^ (28.6% versus 19.5%). Compared with IMF-involved deaths without xylazine, a higher proportion of IMF-involved deaths with xylazine detected were in the Northeast U.S. Census Bureau region (49.9% versus 28.5%) and a lower proportion were in the West (1.1% versus 16.5%).

## Discussion

This report highlights four findings related to detection of xylazine in IMF-involved deaths. First, the percentage of IMF-involved deaths with xylazine detected increased 276% from January 2019 to June 2022. Second, xylazine was detected in <12.0% of IMF-involved deaths overall, but varied by jurisdiction from none to 27.7%, and was highest in the Northeast. Third, when xylazine was detected, whether it was determined to cause death varied by jurisdiction. Finally, demographic characteristics, drug co-involvement, and circumstances were largely similar among IMF-involved deaths with and without xylazine detected.

The timing and magnitude of increase in detection of xylazine among IMF-involved deaths might reflect both increased frequency of testing and true increased presence in the drug supply in recent years^§§§^^,^^¶¶¶ ^(*5*,*6*); however, because of inconsistent testing, detection is still likely underestimated. In April 2023, the White House Office of National Drug Control Policy designated fentanyl adulterated with xylazine an emerging threat****; some jurisdictions have scheduled or are trying to schedule xylazine as a controlled substance.^††††^ Because replacement by similar substances or analogs has previously followed scheduling of certain substances (*7*), monitoring xylazine trends and other sedatives such as medetomidine (*8*) that have recently appeared in the drug supply is important.

The observed geographic variation in xylazine detection could reflect differences in postmortem toxicology testing protocols as well as its varying presence in regional drug supplies. Detection of xylazine and its co-involvement in IMF-involved deaths were most frequent in the Northeast, where IMF-involved deaths increased earlier (*9*) and IMFs are predominantly found in powdered form (*10*). Xylazine detection was lowest in the West, where IMF-involved deaths increased later (*9*) and IMFs are more commonly found in counterfeit pills (*10*). The Drug Enforcement Administration reported that in 2022, 23% of seized fentanyl powder and 7% of seized fentanyl pills contained xylazine.^§§§§^ More information about relative prevalence of xylazine in different forms of IMF products could help tailor overdose prevention efforts to persons using various forms of IMFs.

Decedent demographics, drug co-involvement in death, and overdose circumstances were largely similar among IMF-involved deaths with and without xylazine detected, which suggests that particular demographic groups are not disproportionately affected by xylazine, and certain drugs are not more often used with IMF products with versus without xylazine. Further investigation of whether persons who use drugs are aware of xylazine presence in their products, and motivations for seeking it out or avoiding it, could help tailor prevention messaging.

Among IMF-involved overdose deaths in which xylazine was detected, the percentage for which xylazine was listed as a cause of death ranged from none to ≥90% among jurisdictions. Medical examiners and coroners might differ regarding whether they consider xylazine to increase fatal overdose risk, or they might be unfamiliar with xylazine and therefore not list it on death certificates. This variation highlights the importance of collecting postmortem toxicology data on all drugs detected in overdose deaths, rather than just those listed on the death certificate, especially for emerging drugs. Although health consequences of xylazine in humans are unclear, xylazine can act as a central nervous system depressant and is hypothesized to potentiate sedative effects of opioids (*3*). In this report, xylazine detection was not associated with higher proportions of naloxone administration (among decedents, this would indicate naloxone was administered but failed to reverse the overdose and prevent death) or decedents having no pulse (therefore less likely to respond to rescue measures) when first responders arrived. Further information is needed to understand xylazine’s impact on overdose. Although xylazine has no known antidote and naloxone cannot reverse xylazine-related sedation (*4*), naloxone should be administered to reverse effects of opioids even if xylazine is suspected to be present because xylazine is mainly found in IMF products, which do respond to naloxone. Additional medical care should be sought immediately if overdose involving opioids, xylazine, or both is suspected. Respiratory and cardiovascular support can help address the nonopioid sedative effects of xylazine.

Limited studies indicate that repeated xylazine injections are associated with skin lesions, ulcerations, and abscesses (*4*), suggesting potential long-term xylazine-associated morbidity. Injection drug use evidence was slightly higher among IMF-involved deaths with versus without detection of xylazine; prevention messages could be tailored to persons who inject drugs to promote safe injection practices and proper wound management. Furthermore, harm reduction measures such as using xylazine test strips have shown high efficacy in detecting xylazine in drug products,^¶¶¶¶^ and in conjunction with fentanyl test strips, could inform persons about contents of drug products. This might prevent morbidity or mortality if further evidence supports reports that xylazine contributes to skin lesions or increases overdose risk.

The findings in this report are subject to at least two limitations. First, because analyses were not nationally representative, results might not be generalizable. Second, toxicological testing of xylazine in decedents was not uniform across jurisdictions or over time, likely underestimating the true prevalence of xylazine in overdose deaths, and potentially overestimating the true increase during the analysis period.

Routine toxicology testing for xylazine in suspected overdose cases is critical for accurate surveillance, and further investigation of xylazine’s potency and effects on humans is needed to clarify morbidity and overdose risks and to guide prevention and response efforts. Insight into motivations for adding xylazine to IMF products, and whether persons actively seek xylazine, could help anticipate future drug supply changes and tailor prevention and response efforts. Examining overdose mortality data in conjunction with other data sources, such as drug seizure and nonfatal overdose data, could provide further information about short-and long-term effects of xylazine use.

**Summary**

**What is already known about this topic?**

Xylazine, a nonopioid sedative, has been increasingly detected in illicitly manufactured fentanyl (IMF) drug products and overdose deaths.

**What is added by this report?**

Among 21 jurisdictions, the monthly percentage of IMF-involved deaths with xylazine detected increased 276% from January 2019 (2.9%) to June 2022 (10.9%). During January 2021–June 2022 in 32 jurisdictions, xylazine was detected in a higher percentage of IMF-involved deaths in the Northeast U.S. Census Bureau region; listing xylazine as cause of death varied across jurisdictions.

**What are the implications for public health practice?**

Routine xylazine testing in suspected overdose deaths is critical for surveillance; further investigation of xylazine’s effects on humans is needed to guide prevention efforts. Overdose prevention and response messages should emphasize the need to seek treatment beyond naloxone administration.

## Footnotes

*
https://www.dea.gov/sites/default/files/2022-12/The%20Growing%20Threat%20of%20Xylazine%20and%20its%20Mixture%20with%20Illicit%20Drugs.pdf

†
https://hip.phila.gov/document/2524/PDPH-HAN_Alert_1_Xylazine_03.16.2022.pdf

§ A drug was considered involved or co-involved if it was listed as a cause of death on the death certificate or in the medical examiner or coroner report. Fentanyl was classified as likely illicitly manufactured using toxicology, scene, and witness evidence. For the 8% of deaths involving fentanyl that had insufficient evidence for classification as illicit or prescription, fentanyl was classified as illicit because the vast majority of fentanyl overdose deaths involve illicit fentanyl. All fentanyl analogs except alfentanil, remifentanil, and sufentanil, which have legitimate human medical use, were included as IMFs.

¶ Analyses were restricted to IMF-involved deaths because xylazine is known to be mixed mainly in IMF products and because 99.5% of the deaths in the current analysis with xylazine detected involved IMFs. Among the 42 deaths that did not involve IMFs, IMFs were detected but not part of the cause of death in 17, and not detected in 25. Xylazine was the only drug listed as involved for four deaths.

** Connecticut, Delaware, District of Columbia, Georgia, Illinois, Maine, Massachusetts, Minnesota, Nevada, New Hampshire, New Jersey, New Mexico, Ohio, Oklahoma, Pennsylvania, Rhode Island, Utah, Vermont, Virginia, Washington, and West Virginia. Illinois, Pennsylvania, and Washington reported deaths from counties that accounted for ≥75% of drug overdose deaths in the state in 2017 per SUDORS funding requirements; all other jurisdictions reported deaths from the full jurisdiction. Four funded states that would have been included in the analysis were excluded because programmatic data indicated they were not testing for xylazine during the analysis period. Unpublished programmatic data indicate that xylazine testing was not necessarily uniform within each jurisdiction or over time during the analysis period (e.g., some counties initiated testing sooner than others and testing protocols varied for which cases received xylazine testing).

†† Arizona, Arkansas, Colorado, Connecticut, Delaware, District of Columbia, Georgia, Illinois, Iowa, Kansas, Louisiana, Maine, Maryland, Massachusetts, Michigan, Minnesota, Nebraska, Nevada, New Hampshire, New Jersey, New Mexico, Ohio, Oklahoma, Oregon, Pennsylvania, Rhode Island, South Dakota, Utah, Vermont, Virginia, Washington, and West Virginia. Illinois, Louisiana, Pennsylvania, and Washington reported deaths from counties that accounted for ≥75% of drug overdose deaths in the state in 2017, per SUDORS funding requirements; all other jurisdictions reported deaths from the full jurisdiction. Analyses of overdose circumstances were further restricted to jurisdictions with coroner or medical examiner reports available for ≥75% of deaths in the included periods (this resulted in the same 32 jurisdictions) and to decedents with an available coroner or medical examiner report (52,684). Four funded states that would have been included in analyses were excluded because programmatic data indicated they were not testing for xylazine during the analysis period. Unpublished programmatic data indicate that xylazine testing was not necessarily uniform within each jurisdiction or over time during the analysis period (e.g., some counties initiated testing sooner than others and testing protocols varied for which cases received xylazine testing).

§§ *Northeast:* Connecticut, Maine, Massachusetts, New Hampshire, New Jersey, Pennsylvania, Rhode Island, and Vermont; *Midwest:* Illinois, Iowa, Kansas, Michigan, Minnesota, Nebraska, Ohio, and South Dakota; *South:* Arkansas, Delaware, District of Columbia, Georgia, Louisiana, Maryland, Oklahoma, Virginia, and West Virginia; *West:* Arizona, Colorado, Nevada, New Mexico, Oregon, Utah, and Washington.

¶¶ 45 C.F.R. part 46.102(l)(2), 21 C.F.R. part 56; 42 U.S.C. Sect. 241(d); 5 U.S.C. Sect. 552a; 44 U.S.C. Sect. 3501 et seq.

*** Toxicology report data were not available for ≥75% of all overdose deaths in eight states with complete death certificate data for January 2021–June 2022, and these states were excluded from analyses, but death certificate data identified IMF-involved deaths with xylazine co-involved (indicating that it was detected by postmortem toxicology testing even though the toxicology reports were not submitted to SUDORS): Alabama (46 deaths), Florida (261), Indiana (82), Mississippi (10), Missouri (93), New York (735), South Carolina (178), and Tennessee (167).

††† Route of drug use cannot be directly linked to specific drugs if more than one drug was detected and more than one route reported (e.g., if there was evidence of injection and snorting, both would be documented; if more than one drug was detected, it cannot be determined which was injected and which was snorted).

§§§
https://www.dea.gov/sites/default/files/2022-12/The%20Growing%20Threat%20of%20Xylazine%20and%20its%20Mixture%20with%20Illicit%20Drugs.pdf

¶¶¶ https://www.accessdata.fda.gov/cms_ia/importalert_1179.html (Accessed May 24, 2023).

****
https://www.whitehouse.gov/ondcp/briefing-room/2023/04/12/biden-harris-administration-designates-fentanyl-combined-with-xylazine-as-an-emerging-threat-to-the-united-states/

†††† https://agri.ohio.gov/home/news-and-events/all-news/xylazine-drug-veterinarians-controlle-substance-ohio; https://www.governor.pa.gov/newsroom/governor-shapiro-directs-administration-to-schedule-xylazine-as-a-controlled-substance-taking-action-against-dangerous-drug-contributing-to-opioid-overdoses/

§§§§
https://www.dea.gov/alert/dea-reports-widespread-threat-fentanyl-mixed-xylazine

¶¶¶¶
https://www.cfsre.org/images/content/reports/drug_checking/CFSRE_Xylazine_Report-Rev-1-18-23.pdf
